# 

**Published:** 1903-12

**Authors:** 


### INDEX TO VOLUME XXIV.

A

Academy of Stomatology, 97, 193, 285, 455, 536, 710, 944
Adams, G. E., Treatment of deciduous molars, 927
Address of the chairman of the Section on Stomatology, American Medical Association, 449
After six years of dentistry, 781
"Alba" dentists convicted, 643
American Academy of Dental Science, 27
  Dental Society of Europe, 327
  Medical Association, Section on Stomatology, 33, 326, 677
  Society of Orthodontists, 160, 647, 728, 912
Anderson, Martha, Notes on pulp technique, 446
Angle, Edward H., Some basic principles in orthodontia, 729
A platinum and porcelain crown found useful in certain cases, 796
Arrington, B. F., The possibilities of the tooth-brush, 435
Articulation and articulators, 306
Asepsis and the sterilization of instruments, 338
Asheville, 720
A step backward, 724
Avoid the road to the poor-house, 639

B

Baked porcelain restorations of broken bridge facings, 409
Baker, George T., Exostosis, with loss of tooth: a case in practice, 425
Baldwin, A. E., Professional responsibility, 673
Belden, H. E., Methods of controlling hemorrhage of the oral cavity, 502
BIBLIOGRAPHY:
  A Brief Manual of Prescription Writing in Latin or English. By M. L. Neff, 477
  A Dictionary of Medical Science. By Robley Dunglison, M.D., LL.D., 908
  A Manual of the Injuries and Surgical Diseases of the Face, Mouth, and Jaws. By John Sayre Marshall, 152
  Anatomy and Histology of the Mouth and Teeth. By I. Norman Broomell, 73
  A Pocket Text-Book of Anatomy. By Wm. H. Rockwell, Jr., 316
  Archinard's Bacteriology. By P. E. Archinard, M.D., 963
  How to succeed in the Practice of Medicine. By Joseph McDowell Mathews, 150
  Long's Dental Materia Medica and Therapeutics. By Eli H. Long, 313
  Modern Materia Medica and Therapeutics. By A. A. Stevens, A.M., M.D., 964

Bibliography:
Orthodontia. By J. N. MacDowell, 476
Preface and Summary of the Work of Dr. C. E. de M. Sajous on the Ductless Glands, 317
Principles and Practice of crowning Teeth. By Hart J. Goslee, 961
Principles and Practice of filling Teeth. By C. N. Johnson, 394
Success in Dental Practice. By C. N. Johnson, 558
Syphilis in Dentistry. By L. Blake Baldwin, M.D., 809
The Care of the Teeth. By Samuel A. Hopkins, 74
The Filling of Teeth with Porcelain (Jenkins's System). By Walter Wolfgang Bruck, 557, 810
The Internal Secretions and the Principles of Medicine. By Charles E. de M. Sajous, 472
Transactions of the American Röntgen Ray Society, 559
Bogue, E. A., Some of the causes of irregular teeth, with suggestions as to preventive treatment or early cure, 241
The principal molar in man, and its relations to, and bearings upon, the other teeth, 569
Brackett, Charles A., Our sayings, 17
Brewster, Robert, What place has low-fusing porcelain in dentistry? 433
Bryan, L. C., Locating and curing abscess tracts, 938
Prophylaxis: extension *for* prevention, or extension *of* prevention, 831
Treating sensitive dentine, 937

C

Central Dental Association of Northern New Jersey, 408
Chaddock, Charles Gilbert, Pain, 613
Chittenden, Chas. C., Is the realization of reasonable ideals in dental education near at hand? 525
Cleansing the oral cavity, 935
Committees of fifteen and nine, the, 555
Connecticut State dental commissioners, 568, 822
Conservatism *vs.* radicalism in certain dental operations, 171
Constant, Thos. E., The dental pulp, viewed without the microscope, 411
Consul-General Worman's correspondence, 157
Convention period, the, 551
Correction, 313
Current News:
American Dental Society of Europe, 327
Medical Association, Section on Stomatology, 326
Society of Orthodontists, 160, 647, 728, 912
Central Dental Association of Northern New Jersey, 408
Connecticut State dental commissioners, 568, 822
Fourth International Dental Congress, 819, 972
Golden anniversary celebration, 975
Hartford Dental Society, 976
Harvard Dental Alumni Association, 648
Odontological Society, 487

Current News:
Illinois State Dental Society, 405, 568
Institute of Dental Pedagogics; 240, 823, 911, 974
Kentucky State Dental Association, 328, 406
Lebanon Valley Dental Association, 406
Massachusetts Dental Society, 328, 408
Minnesota State Dental Association, 648
Mississippi Board of Dental Examiners, 406
Missouri State Dental Association, 407, 647
National Association of Dental Examiners, 823
of Dental Faculties, 487
Dental Association, 486
Nebraska State Dental Society, 328, 408
New Jersey State Board of Registration and Examination in Dentistry, 405, 728
State Dental Society, 486
New Orleans Academy of Stomatology, 407
Northeastern Dental Association, 824
Odontographic Society of Chicago, 160
Pennsylvania Board of Dental Examiners, 971
State Dental Society, 406, 486
Report of the committee on congresses; Fourth International Dental Congress, St. Louis, 1904, 563
Seventh and Eighth District Dental Societies of New York, 824
South Dakota Dental Society, 488
Tennessee Dental Association, 567
The Fraternal Dental Society of St. Louis, Mo., 912
The New York Institute of Stomatology, 240
Wisconsin State Dental Society, 488
Curry, John C., Some considerations pertaining to dental diagnosis, 349
Custer, L. E., Practical use of the X-ray in dentistry, 247

D

Davenport, William Slocum, Dental bridge and pier construction, 161
Deciduous molars, treatment of, 927
Degree of D.D.S., origin of, 155
Dental bridge and pier construction, 161
diagnosis, some considerations pertaining to, 349
hospital, Sydney, New South Wales, 71
notes, 168
pulp, the, viewed without the microscope, 411
regulations in the British Isles, 15
Domestic Correspondence:
Honor to Dr. Jenkins, 966
Origin of the degree of D.D.S., 155
Rainy-day thoughts, 318
Resolution on interchange of license, 811
The older methods of filling teeth, 478

Dray, Arthur R., Regeneration of the pulp, 81
Duffield, Joseph E., Baked porcelain restorations of broken bridge facings, 409

E

Eaton, Horace E., Management of children in the dental office, 8
Editorial:
"Alba" dentists convicted, 643
Asheville, 1903, 720
A step backward, 724
Avoid the road to the poor-house, 639
Correction, 313
Dental hospital, Sydney, New South Wales, 71
Errors in expression and practice, 235
Has the art of filling teeth progressed in fifty years? 309
Importance of analytical thinking, 389
Institute of Dental Pedagogics, 958
Interstate reciprocity, 468
Is the eruption of teeth caused by blood-pressure? 804
J. Foster Flagg, D.D.S., 959
Jonathan Taft, M.D., D.D.S., 906
Medical reciprocity, 643
Memorial window to Horace Wells, 393
New York Regents and the D.D.S. degree, 71
Recognition of the D.D.S. degree by the American Medical Association, 556
Salutation, 66
Stewart B. Palmer, M.D.S., 391
The committees of fifteen and nine, 555
The convention period, 551
The dangers of paternalism, 139
The fifty per cent. resolution of "examiners," 905
The Fourth International Dental Congress, 1904, 954
The organization of international dental congresses, 141
The outlook for the future, 68
The souvenir medal of the Fourth International Dental Congress, 957
United States Consul-General J. H. Worman, 149
W. C. Barrett's "link in our professional history," 898
William Cary Barrett, 807
Eglin, A. C., The removable bridge as a conservator of teeth, 663
Empyema of the antrum, 608
English tube-teeth, some uses for, 1
Errors in expression and practice, 235
Expansion of gutta-percha, 633
Exostosis, with loss of tooth: a case in practice, 425

F

Fernald, Adelbert, The first men to practise dentistry in Boston, 511
Fifty per cent. resolution of "examiners," 905

First men to practise dentistry in Boston, 511
Flagg, J. Foster, D.D.S., 959
Fletcher, M. H., Tolerance of the tissues to foreign bodies, with special reference to the pulp and gums, 596
FOREIGN CORRESPONDENCE:
Consul-General Worman's correspondence, 157
Dr. M. H. Cryer's visit to Dublin, Ireland, 811
Letter from Dr. N. S. Jenkins, Dresden, Germany, 560
Fourth International Dental Congress, 819, 954, 972
Fraternal Dental Society of St. Louis, Mo., 912

G

Giant sarcoma, report of a case of, 86
Glazier, C. M., Seamless gold crowns, 796
Gold fillings, 936
Golden anniversary celebration, 975

H

Harrison, Walter, Dental regulations in the British Isles, 15
Hartford Dental Society, 976
Harvard Dental Alumni Association, 648
Odontological Society, 121, 487
Has the art of filling teeth progressed in fifty years? 309
Heise, O. N., Empyema of the antrum, 608
Hemorrhage of the oral cavity, methods of controlling, 502
Holden, George M., Importance of organized efforts, 87
Honor to Dr. Jenkins, 966
Hopkins, Samuel A., Asepsis and the sterilization of instruments, 338
Medical aspects of dental lesions, 576
How far do stomatologic indications warrant constitutional treatment? 347
Howe, J. Morgan, Some useful analgesics, 260
Percy R., Cases of maxillary sinus derangement, 913
Human dental pulp, self-healing capacity of, 649

I

Illinois State Dental Society, 405, 568
Importance of analytical thinking, 389
of the mesio-distal relation of the two arches during childhood, 842
of organized efforts, 87
INCIDENTS OF PRACTICE:
A new obtundent, 322
Beta-eucaine, 320
Carbolic acid as a coagulator, 321
Deaths from swallowing false teeth, 322
Dovetail porcelain inlays, 323
Gutta-percha for setting inlays, 322
Oil of cloves, 239
Observation of case of resorption reported on page 633 of the INTERNATIONAL DENTAL JOURNAL for 1902, 239

INCIDENTS OF PRACTICE:
Sensitive cervical cavities, 397
Traumatic impaction of both deciduous centrals, 239
Treatment of a case of "dry socket," 397
of putrescent pulp-canals, 320
Influence of diseases of the air-passages on the teeth, 670
Institute of Dental Pedagogics, 240, 823, 911, 958, 974
Interchange of license, resolution on, 811
International dental congresses, the organization of, 141
Dental Federation and International Commission of Education, 51, 221, 371, 461, 547
Interstate reciprocity, 468
Irregular teeth, some of the causes of, with suggestions as to preventive treatment or early cure, 21
Is the eruption of teeth caused by blood-pressure? 804

J

Jenkins, N. S., The making of a porcelain filling, 426
Jumping the bite, 594

K

Kells, C. Edmund Jr., What is the meaning of conservatism in dental practice? 532
Kentucky State Dental Association, 328, 406

L

Lebanon Valley Dental Association, 406
Letter from Dr. N. S. Jenkins, Dresden, Germany, 560
"Link in our professional history," W. C. Barrett's, 898
Locating and curing abscess tracts, 938
Longevity and efficiency, 839
Looking backward and forward, 261
Luckie, S. Blair, Cleansing the oral cavity, 935

M

McCullough, P. B., The round vacuum chamber, 850
Management of children in the dental office, 8
Marvel, William W., Longevity and efficiency, 839
Massachusetts Board of Registration in Dentistry—Resolutions of respect, 78
Dental Society, 42, 103, 202, 293, 328, 408, 704, 860
Maxillary sinus derangement, cases of, 913
Meaning of conservatism in dental practice, 532
Medical and dental libraries, 604
aspects of dental lesions, 576
reciprocity, 643
Memorial window to Horace Wells, 393
Meriam, Horatio C., Some uses for English tube-teeth, 1
Midgley, Albert L., Noma, with report of a case, 422

Miller, W. D., The self-healing capacity of the human dental pulp, 649
Mills, G. Alden, Looking backward and forward, 261
Minnesota State Dental Association, 648
Minshall, A. G., The influence of diseases of the air-passages on the teeth, 670
MISCELLANY:
Adrenalin in devitalization of pulps, 645
A fossil unearthed in Austria, 324
A method for backing porcelain teeth for metal work, 480
A new antiseptic, 485
A new local anæsthetic discovered, 482
An oxyacetylene blow-pipe, 325
A suggestion, 480
Beeswax for filling roots, 817
Bleaching an anterior tooth, 484
Care of the hands, 817
Celluloid dentures, 562
splint for supporting loose teeth, 727
Cement anchorage for amalgam fillings, 483
Chemical test for sodium dioxide, 325
Chipping of porcelain teeth in soldering, 562
Chloroform liniment for dental use, 816
Cleansing the hands, 819
Coloring artificial teeth, 482
Crystallized peroxide of hydrogen, 479
Definition for articulation, 482
Dental inspection in schools, 484
Extractions of canines, 647
Formaldehyde for pulp-canal cleansing, 816
Hemorrhagic diathesis controlled by calcium chloride, 726
Manipulation and care of cements, 646
Neurasthenic neuralgias, 481
Polishing porcelain, 483
Salol as a mouth-wash, 481
Sensitive necks of teeth, 647
Sharpening lancets, excavators, etc., 818
The Sanger half-collar crown, 818
To perfectly fit clasps, 325
Mississippi Board of Dental Examiners, 406
Missouri State Dental Association, 407, 647
Mummification of the dental pulp, notes on, 251

### N

National Association of Dental Examiners, 823
of Dental Faculties, 487
Dental Association, 486
Neall, Walter H., Notes on mummification of the dental pulp, 251
Nebraska State Dental Society, 328, 408

New Jersey State Board of Registration and Examination in Dentistry, 405, 728
State Dental Society, 486, 894
New Orleans Academy of Stomatology, 407
New York Regents and the D.D.S. degree, 71
Noma, with report of a case, 422
Northeastern Dental Association, 824

O

OBITUARY:
Barrett, William C., M.D., D.D.S., M.D.S., 813
Hurlbut, Dr. J. Searle, 79
Palmer, Stewart Bailey, M.D.S., 398
Resolutions of respect to Dr. J. Searle Hurlbut, 77, 78
Resolutions of respect to Dr. Jonathan Taft, 971
Smyth, W. J., M.D.S., L.D.S., 324
Taft, Jonathan, M.D., D.D.S., 966
Woodward, Dr. Corydon A., 644
Obligations of the dentist to himself, suggestions regarding, 930
Odontographic Society of Chicago, 160
Older methods of filling teeth, 478
ORIGINAL COMMUNICATIONS:
Address of the chairman of the Section on Stomatology, American Medical Association, 449
After six years of dentistry, 781
A platinum and porcelain crown found useful in certain cases, 796
Asepsis and the sterilization of instruments, 338
Baked porcelain restorations of broken bridge facings, 409
Cases of maxillary sinus derangement, 913
Cleansing the oral cavity, 935
Conservatism *vs.* radicalism in certain dental operations, 171
Dental bridge and pier construction, 161
Dental notes, 168
Dental regulations in the British Isles, 15
Empyema of the antrum, 608
Exostosis, with loss of tooth: a case in practice, 425
Expansion of gutta-percha, 633
Gold fillings, 936
How far do stomatologic indications warrant constitutional treatment? 347
Importance of organized efforts, 87
Is the realization of reasonable ideals in dental education near at hand? 524
Jumping the bite, 594
Longevity and efficiency, 839
Looking backward and forward, 261
Management of children in the dental office, 8
Medical and dental libraries, 604

ORIGINAL COMMUNICATIONS:

Medical aspects of dental lesions, 576
Methods of controlling hemorrhage of the oral cavity, 502
Noma, with report of a case, 422
Notes on mummification of the dental pulp, 251
Notes on pulp technique, 446
Our sayings, 17
Pain, 613
Points of interest in a twenty years' practice, 666
Porcelain, 329
  a fad or a fact, 847
  inlays, 255
    and restorations, 489
Practical use of the X-ray in dentistry, 247
Professional responsibility, 673
Prophylaxis: extension *for* prevention, or extension *of* prevention? 831
Pulp hypertrophy of the teeth, 442
Pyorrhœa alveolaris: its causes and treatment, 268
Recognition of the D.D.S. degree by the American Medical Association, 529
Report of a case of giant sarcoma, 86
Sanitalogy: a discussion of the health principle, 769
Seamless gold crowns, 797
Septic infection from a tooth, 846
Some basic principles in orthodontia, 729
Some considerations pertaining to dental diagnosis, 349
Some of the causes of irregular teeth, with suggestions as to preventive treatment or early cure, 241
Some useful analgesics, 260
Some uses for English tube-teeth, 1
Suggestions regarding the obligations of the dentist to himself, 930
Syphilitic interstitial gingivitis, 85
The dental pulp, viewed without the microscope, 411
The first men to practise dentistry in Boston, 511
The importance of the mesio-distal relation of the two arches during childhood, 842
The influence of diseases of the air-passages on the teeth, 670
The making of a porcelain filling, 426
The porcelain art in dentistry, 825
The possibilities of the tooth-brush, 435
The principal molar in man, and its relations to, and bearings upon, the other teeth, 569
The regeneration of the pulp, 81
The removable bridge as a conservator of teeth, 663
The round vacuum chamber, 850
The self-healing capacity of the human dental pulp, 649
The technique of approximal restorations with gold in posterior teeth, 652

Original Communications:
Tolerance of the tissues to foreign bodies, with special reference to the pulp and gums, 596
Treatment of deciduous molars, 927
"We made better dentists then than now," 917
What is the meaning of conservatism in dental practice? 532
What place has low-fusing porcelain in dentistry? 433
Why dentists do not read, 258
Winning the confidence of children, 91
Our sayings, 17
Outlook for the future, 68

P

Pain, 613
Palmer, J. G., Conservatism *vs.* radicalism in certain dental operations, 171
Parkhurst, Charles E., After six years of dentistry, 781
Paternalism, the dangers of, 139
Patten, Charles C., The porcelain art in dentistry, 825
Pennsylvania Board of Dental Examiners, 971
State Dental Society, 406, 486
Piper, James R., Septic infection from a tooth, 846
Points of interest in a twenty years' practice, 666
Porcelain, 329
a fad or a fact, 847
art in dentistry, 825
filling, the making of a, 426
inlays, 255
and restorations, 489
Possibilities of the tooth-brush, 435
Preparing teeth for histological examination, some methods of, 22
Principal molar in man, the, and its relations to, and bearings upon, the other teeth, 569
Professional responsibility, 673
Prophylaxis: extension *for* prevention, or extension *of* prevention? 831
Pulp hypertrophy of the teeth, 442
technique, notes on, 446
Pyorrhœa alveolaris, its causes and treatment, 268

R

Rainy-day thoughts, 318
Realization of reasonable ideals in dental education, 525
Recognition of the D.D.S. degree by the American Medical Association, 529, 556
Reeves, W. T., Porcelain inlays and restorations, 489
Regeneration of the pulp, 81
Removable bridge as a conservator of teeth, 663
Report of the committee on congresses: Fourth International Dental Congress, St. Louis, 1904, 563

REPORTS OF SOCIETY MEETINGS:
Academy of Stomatology, 97, 193, 285, 455, 536, 710, 944
American Academy of Dental Science, 27
Medical Association, Section on Stomatology, 33, 677
Harvard Odontological Society, 121
International Dental Federation and International Commission of Education, 51, 221, 371, 461, 547
Massachusetts Dental Society, 42, 103, 202, 293, 704, 860
New Jersey State Dental Society, 894
New South Wales Dental Defence Association, 64
Tennessee Dental Association, 953
The New York Institute of Stomatology, 178, 281, 361, 634, 799, 852, 937
REVIEWS OF DENTAL LITERATURE:
Abstract from an interesting paper on the therapy of various forms of light and radio-therapy, 357
A simple and improved method of taking an impression with plaster, 360
Dental use of homœopathic remedies, 274
Further investigations in regard to " the saliva as a natural protection against caries," 454
Mechanics of the jaws, 276
New method of devitalizing the pulp, 360
Paramidobenzoic acid, 96
Some methods of preparing teeth for histological examination, 22
Somnoform, an anæsthetic, 270
Rhein, M. L., Address of the chairman of the Section on Stomatology, American Medical Association, 449
The technique of approximal restorations with gold in posterior teeth, 652
Robinson, J. Arthur, Articulation and articulators, 306
Rollins, William, Dental notes, 168
Ross, James, Porcelain: a fad or a fact, 847
Round vacuum chamber, the, 850

S

Salutation, 66
Sanitalogy: a discussion of the health principle, 769
Seamless gold crowns, 797
Septic infection from a tooth, 846
Smith, F. Milton, Suggestions regarding the obligations of the dentist to himself, 930
Murdock C., Report of a case of giant sarcoma, 86
T. Victor, Porcelain inlays, 255
Some basic principles in orthodontia, 729
Some useful analgesics, 260
South Dakota Dental Society, 488
Souvenir medal of the Fourth International Dental Congress, 957
Stanley, Ned A., A platinum and porcelain crown found useful in certain cases, 796

Stanley, Ned A., Points of interest in a twenty years' practice, 666
Steeves, Alice M., Medical and dental libraries, 604
Stewart B. Palmer, D.D.S., 391
Stubblefield, D. R,, Sanitalogy: a discussion of the health principle, 769
"We made better dentists then than now," 917
Syphilitic interstitial gingivitis, 85

T

Taft, Jonathan, M.D., D.D.S., 906
Talbot Eugene S., How far do stomatologic indications warrant constitutional treatment? 347
Recognition of the D.D.S. degree by the American Medical Association, 529
Syphilitic interstitial gingivitis, 85
Why dentists do not read, 258
Taylor, Frank T., Expansion of gutta-percha, 633
Technique of approximal restorations with gold in posterior teeth, 652
Tennessee Dental Association, 567, 953
Terrell, George B., Jumping the bite, 594
The New York Institute of Stomatology, 178, 240, 281, 361, 634, 799, 852, 937
Tolerance of the tissues to foreign bodies, with special reference to the pulp and gums, 596
Treating sensitive dentine, 937
Trostil, H. Elmer, Gold fillings, 936

U

United States Consul-General J. H. Worman, 149

V

Valley District Dental Society—Resolutions of respect, 77
Visit to Dublin, Ireland, Dr. M. H. Cryer's, 811
Von Römer, Privatdocent Dr., Pulp hypertrophy of the teeth, 442

W

Weeks, S. M., The importance of the mesio-distal relation of the two arches during childhood, 842
"We made better dentists then than now," 917
Wentworth, Evan P., Winning the confidence of children, 91
Wetzel, Eugene J., Pyorrhœa alveolaris: its causes and treatment, 268
What place has low-fusing porcelain in dentistry? 433
Wheeler, Herbert Locke, Porcelain, 329
Why dentists do not read, 258
William Cary Barrett, 807
Winning the confidence of children, 91
Wisconsin State Dental Society, 488

X

X-ray, practical use of, in dentistry, 247

